# Predicting multiple taste sensations with a multiobjective machine learning method

**DOI:** 10.1038/s41538-024-00287-6

**Published:** 2024-07-25

**Authors:** Lampros Androutsos, Lorenzo Pallante, Agorakis Bompotas, Filip Stojceski, Gianvito Grasso, Dario Piga, Giacomo Di Benedetto, Christos Alexakos, Athanasios Kalogeras, Konstantinos Theofilatos, Marco A. Deriu, Seferina Mavroudi

**Affiliations:** 1 InSyBio PC, Patras, 265 04 Greece; 2 PolitoBIOMedLab, Department of Mechanical and Aerospace Engineering, Politecnico di Torino, Torino, 10129 Italy; 3 Industrial Systems Institute, Athena Research Center, 265 04 Patras, Greece; 4 Department of Innovative Technologies, Dalle Molle Institute for Artificial Intelligence, Lugano-Viganello, 6962 Switzerland; 5 Enginlife Engineering Solutions, Turin, Italy; 6 Department of Nursing, University of Patras, 265 04 Patras, Greece

**Keywords:** Chemical engineering, Nutrition, Biomedical engineering, Biochemistry

## Abstract

Taste perception plays a pivotal role in guiding nutrient intake and aiding in the avoidance of potentially harmful substances through five basic tastes - sweet, bitter, umami, salty, and sour. Taste perception originates from molecular interactions in the oral cavity between taste receptors and chemical tastants. Hence, the recognition of taste receptors and the subsequent perception of taste heavily rely on the physicochemical properties of food ingredients. In recent years, several advances have been made towards the development of machine learning-based algorithms to classify chemical compounds’ tastes using their molecular structures. Despite the great efforts, there remains significant room for improvement in developing multi-class models to predict the entire spectrum of basic tastes. Here, we present a multi-class predictor aimed at distinguishing bitter, sweet, and umami, from other taste sensations. The development of a multi-class taste predictor paves the way for a comprehensive understanding of the chemical attributes associated with each fundamental taste. It also opens the potential for integration into the evolving realm of multi-sensory perception, which encompasses visual, tactile, and olfactory sensations to holistically characterize flavour perception. This concept holds promise for introducing innovative methodologies in the rational design of foods, including pre-determining specific tastes and engineering complementary diets to augment traditional pharmacological treatments.

## Introduction

Taste and smell play a pivotal role in the chemosensory perception of food since they are fundamental determinants for the food selection and intake process^1^. Biochemical compounds derived from food ingestion trigger the taste perception process through the binding with specific proteins known as taste receptors, located on the tongue’s taste buds and dedicated to the recognition of the five basic tastes: sweet, bitter, sour, salty, and umami^2,3^. Sweet taste is commonly associated with energy-rich food, to help identify sources of sugars and carbohydrates^4^. Conversely, bitter taste is normally recognized as an unpleasant flavour and acts as a warning against potentially dangerous compounds^5^. Sour taste helps to detect spoiled food and identify the presence of biologically relevant vitamins^6^. Salty taste is crucial to monitor the uptake of essential electrolytes, which play a central role in maintaining body osmosis^7^. Finally, umami taste relates to the protein content in food, through the recognition of amino acids and oligopeptides^8^. Therefore, each taste is associated with critical biological functions and nutritional needs that are important to preserve health status. In this sense, the taste of chemical compounds present in food stimulates an increase in nutrient intake while helping to avoid potentially harmful substances^9^. Indeed, nutritious foods usually have an appetitive taste, e.g., sweet, umami, or a low concentration of salts and acids. Instead, toxic substances usually present a repulsive flavour, such as bitter tastants, strong sour taste stimuli, and high concentrations of salts^10^. In general, taste sensation relies on the affinity of specific biochemical compounds and their target taste receptors^11,12^. Small variations in tastants’ chemistry may result in a drastic change in perceived taste. Therefore, shedding light on the physio-chemical features of compounds in food is of primary importance to pinpoint the molecular bases and mechanisms determining the food taste and subsequent food consumption.

In recent years, several studies have developed machine-learning (ML) tools to predict the taste of specific compounds starting from their chemical structure^13^. In literature, there is a net prevalence of ML tools for predicting sweet and bitter tastes e.g., BitterX^14^, BitterPredict^15^, e-Bitter^16^, iBitter-SCM^17^, BERT4Bitter^18^, iBitter-Fuse^19^, a QSTR-based approach^20^, e-Sweet^21^, Predisweet^22^, BoostSweet^23^, BitterSweetForest^24^, BitterSweet^25^, bitter-sweet classifier by Bo et al^26^., VirtuousSweetBitter^27^. Five examples of umami taste predictors are instead present in recent literature, namely, iUmami-SCM^28^, UMPred-FRL^29^, VirtuousUmami^30^, Umami-MRNN^31^, and Umami-BERT^32^. From a technical point of view, several ML algorithms are used for taste prediction, among which Multiple Linear Regression (MLR) and Support Vector Machine (SVM) were the first used models for binary classification^13,33^. Thanks to recent scientific progress, these models were outclassed by tree-based models, such as Random Forest (RF) or AdaBoost (AB), and Neural Network (NN)^34,35^. Within this framework, the latter algorithms also support multi-class classification and work well in the non-linear classification domain, if the selected database is large enough. Multi-class and multi-labelling techniques have been employed in several applications for the food and agricultural industries, but there are still limited applications for classifying compounds by taste and predicting the relative taste intensity^36–39^. Recently, a multi-class classification method based on learning vector quantization NN to classify tea samples of five commercial brands has been proposed^40^. Monforte et al. presented an orthogonal partial least square discriminant analysis (OPLS-DA) and RF-combined multi-class pipeline for the discrimination of white wine ageing based on target oxidation markers^41^. Moreover, an SVM multiclass classification demonstrated high efficiency in the classification of 7 different types of raw food^42^. Given the aforementioned context, there is a notable lack of research focused on the simultaneous prediction of multiple tastes, especially in real-world scenarios where foods frequently exhibit a complex blend of tastes.

This lack of tools for predicting multiple tastes represents a significant limitation in the field of food science and technology, specifically in the formulation and optimization of food products. In this study, we address these gaps by developing a machine learning-based tool that predicts not one, but four different tastes, and by focusing on the underlying physicochemical properties that contribute to these tastes. In the present work, we aimed at developing a multi-class taste predictor, named VirtuousMultiTaste, able to distinguish between bitter, sweet, and umami from other taste sensations. We employed a hybrid combination of heuristic optimization and nonlinear machine learning classification methods. Building upon our previous work^30^, this new four-taste predictor was trained and tested using similar ensemble dimensionality reduction and classification techniques. This approach effectively reduced the number of physicochemical features and identified the important ones for predicting the four different tastes. The simplicity of the model reduces the likelihood of overfitting and makes it more user-friendly through a web interface (https://virtuous.isi.gr/#/virtuous-multitaste), expanding the potential audience of users. VirtuousMultiTaste is a machine learning-based web tool that predicts four different tastes and opens the possibility of analysing different compounds and understanding the chemical-physical factors that contribute to the overall taste perception.

## Results

### Dimensionality reduction

As outlined in the Methods section, the statistical analysis to reduce the number of employed molecular descriptors was performed on the training set with the Kruskal–Wallis test^43^ since not all features followed normal distribution when tested with the Shapiro–Wilk test^44^. Moreover, the correction of p-values for multiple testing to get q-values was applied using the Benjamini-Hochberg FDR adjustment method^45^. By setting the *q*-value threshold to 0.05, we identified 1306 statistically significant differentiated features. The entire set of molecular features and 1306 statistically significant differentiated ones were also analysed using the Principal Component Analysis (PCA) and represented in reduced dimensional spaces according to the first three principal components (see also Supplementary Fig. 1). The lack of clear separation among tastes in the PCA plots underlined the complexity of the multi-taste prediction problem and highlighted the limitations of linear dimensionality reduction techniques in discriminating between different tastes. This limitation pushes us towards more sophisticated machine learning algorithms. Pairwise comparisons of each taste versus the rest were further conducted using the Mann-Whitney test. Supplementary Fig. 2 presents a heatmap of the top 5 features differentiating each taste from the rest in our training dataset.

### Model performance

**Fig. 1:**
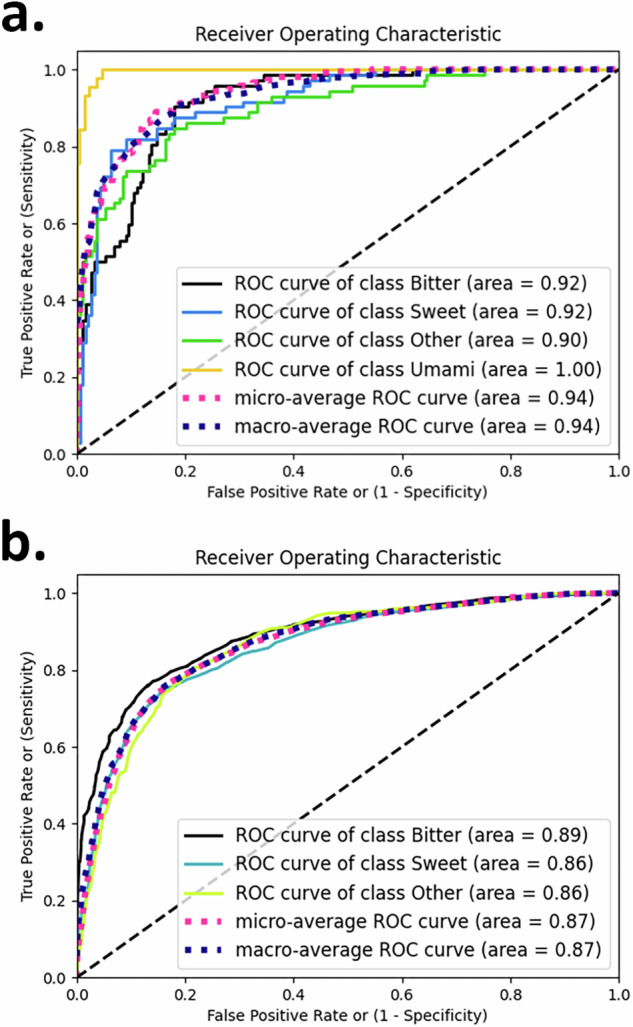
Receiver operating characteristic (ROC) curves. ROCs of the four-taste classification for (**a**) the cross-validation set and (**b**) the testing set.

The multi-objective evolutionary optimization algorithm employed in our study indicated that the RF method achieved better performance across multiple objectives compared to SVM. Therefore, we developed and evaluated 20 different RF models (see also Supplementary Table 1). Among these models, we selected the best model (RF model #2 in Supplementary Table 1) considering the balance between the achieved performance and the relatively small number of features (15). A summary of the model performance is reported in Table 1. During the training phase, we employed 10-fold cross-validation (CV) on the training set, presenting mean values and standard deviations for the performance metrics. The testing set comprises the 3377 left-out samples not used for training, consisting of 1577 bitter, 1544 sweet and 289 other compounds. The relative ROC curves are represented in Fig. 1A. In Table 2 and Table 3, a summary of the performance of each taste class is presented for the training set and the testing set, respectively. The relative ROC curves from the validation of the testing set are represented in Fig. 1B.

**Table 1:** Summary of model performance with the selected RF model in the 10-fold cross-validation (CV) and test sets

	ACC	F1	F2	Precision	Recall	AUC
CV	76.54% ± 1.0	76.58% ± 1.0	76.61% ± 1.01	76.92% ± 1.05	76.64% ± 1.0	0.92 ± 0.02
Test	71.76%	74.32%	73.10%	78.98%	71.76%	0.87

**Table 2:** Summary of the 10-fold cross-validation training performance for each class

	ACC	F1	F2	Precision	Recall	AUC
Bitter	88.56% ± 0.81	79.09% ± 1.78	77.31% ± 3.30	82.42% ± 0.63	77.21% ± 4.19	0.92 ± 0.0.16
Sweet	83.43 ± 0.79	73.26% ± 0.85	75.59% ± 2.23	70.03% ± 1.30	75.65 ± 2.81	0.90 ± 0.01
Other	82.83% ± 0.43	66.62% ± 0.13	64.03% ± 1.27	67% ± 1.39	63.57% ± 1.71	0.86 ± 0.10
Umam**i**	95.99% ± 0.16	88.49% ± 0.51	88.64% ± 0.68	87.61% ± 0.67	87.40% ± 1.17	0.98 ± 0.005

**Table 3:** Summary of model performance using the final trained model for each taste class in the test set

	ACC	F1	F2	Precision	Recall	AUC
Bitter	81.70%	79.01%	76.24%	84.10%	74.50%	0.89
Sweet	79.53%	75.38%	71.57%	82.73%	69.23%	0.86
Other	84.19%	42.96%	56.01%	30.94%	70.24%	0.86

### Feature Importance

**Fig. 2:**
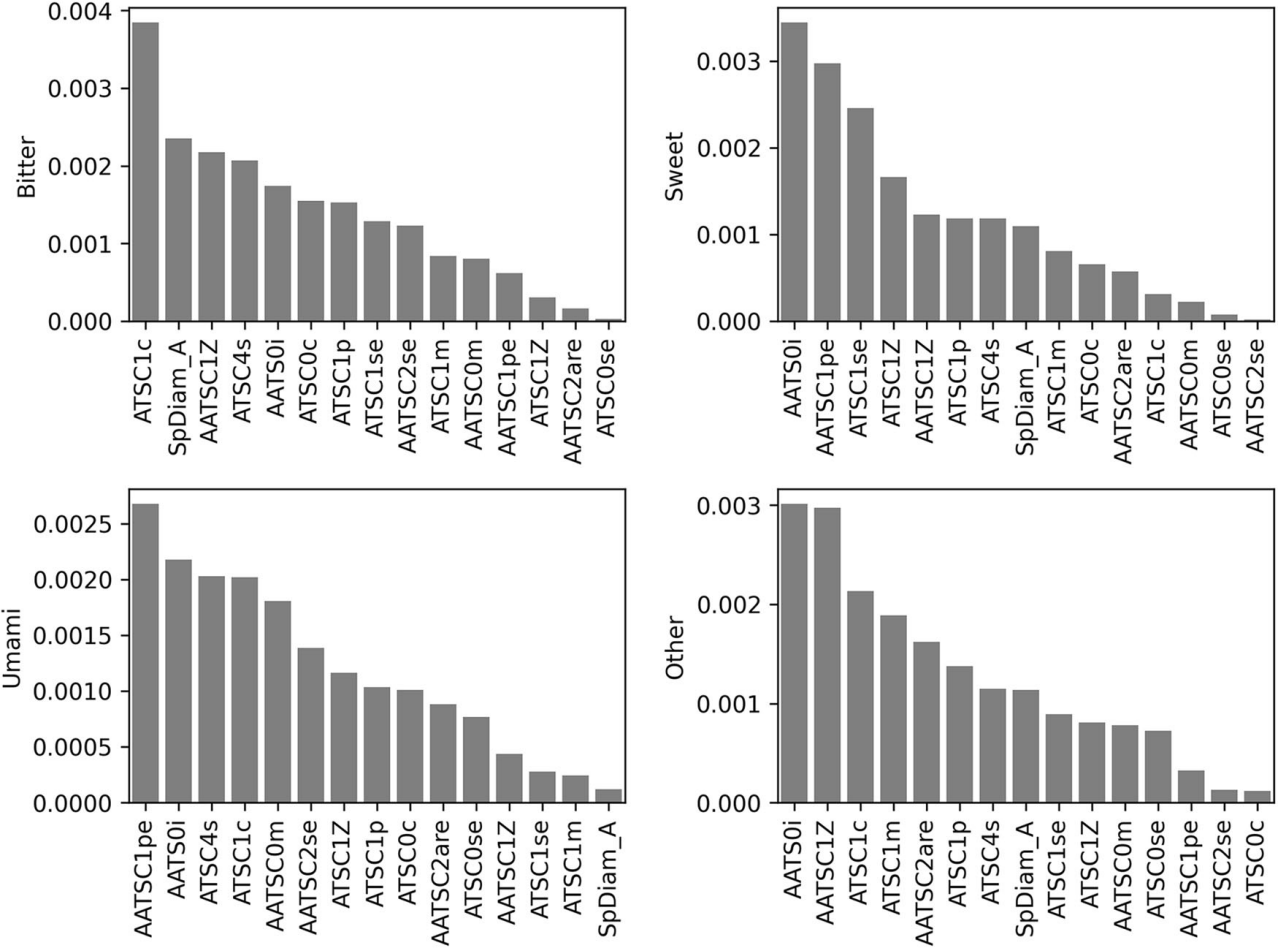
Feature importance using SHAP. Feature importance of the selected features using the average of the absolute SHAP values of each taste class and ranking features in order of importance.

Feature importance is a crucial aspect of ML models as it provides valuable insights into the contribution and relevance of different input features for making accurate predictions. The features selected during the model construction on which the predictions rely are 15 and include the molecular descriptors ATSC0c, ATSC0se, AATS0i, ATSC1p, AATSC2se, AATSC0m, AATSC1Z, AATSC2are, AATSC1pe, SpDiam_A, ATSC1c, ATSC1se, ATSC1Z, ATSC1m, and ATSC4s. The selected features are summarized in Fig. 2 reporting the level of importance according to the SHAP values. The distributions of the 15 features for the bitter, sweet, other and umami samples are also represented in Supplementary Fig. 3.

**Fig. 3:**
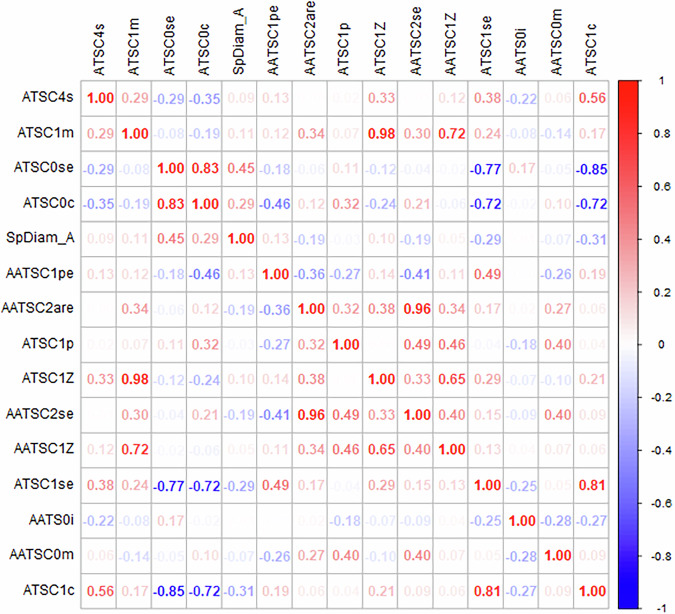
Correlation plot of the best 15 selected features. Each square represents the correlation between two features. The colour and value of each square indicate the magnitude of the correlation coefficient, with blue and red values indicating negative and positive correlations, respectively.

In this connection, the correlation between the selected features holds great importance in understanding the underlying relationships and interactions within the taste prediction model. Figure 3 represents the correlation between the 15 most important features selected by the model during training.

### External datasets

The developed model was tested to screen external datasets collecting food-related and natural compounds:

1. FooDB (https://foodb.ca/) is the most extensive and comprehensive resource worldwide concerning food constituents, chemistry and biology, containing over 70000 compounds. After having removed missing SMILES, duplicate compounds, and molecules with structural errors, we ended up with 69309 molecules: 14693 were predicted as Bitter, 5375 as Sweet, 3149 as Umami and 46092 as Other.
2. FlavorDB (https://cosylab.iiitd.edu.in/flavordb/) collects information regarding flavour molecules. For the present work, we considered only 2599 molecules related to natural ligands from the dataset: 778 were predicted as Bitter, 1661 as Sweet, 29 as Umami and 131 as Other.
3. PhenolExplorer (http://phenol-explorer.eu) is a comprehensive database that compiles information on polyphenols found in foods. We considered only compounds having composition data (SMILES), resulting in 489 compounds: 365 were predicted as Bitter, 23 as Sweet, 9 as Umami and 92 as Other.
4. Natural Product Atlas (https://www.npatlas.org/) encompasses naturally occurring compounds derived from microorganisms, as documented in peer-reviewed primary scientific literature. We preserved 32491 compounds after curating the dataset with the CHEMBL structure pipeline: 26653 were predicted as Bitter, 2019 as Sweet, 1880 as Umami and 1939 as Other.
5. PhytoHub (https://phytohub.eu/) is an openly accessible electronic database containing detailed information about dietary phytochemicals and their metabolites in humans and animals. We preserved 1746 compounds after having removed missing SMILES and compounds with issues in their molecular structures: 1213 were predicted as Bitter, 228 as Sweet, 62 as umami and 243 as Other.

**Fig. 4:**
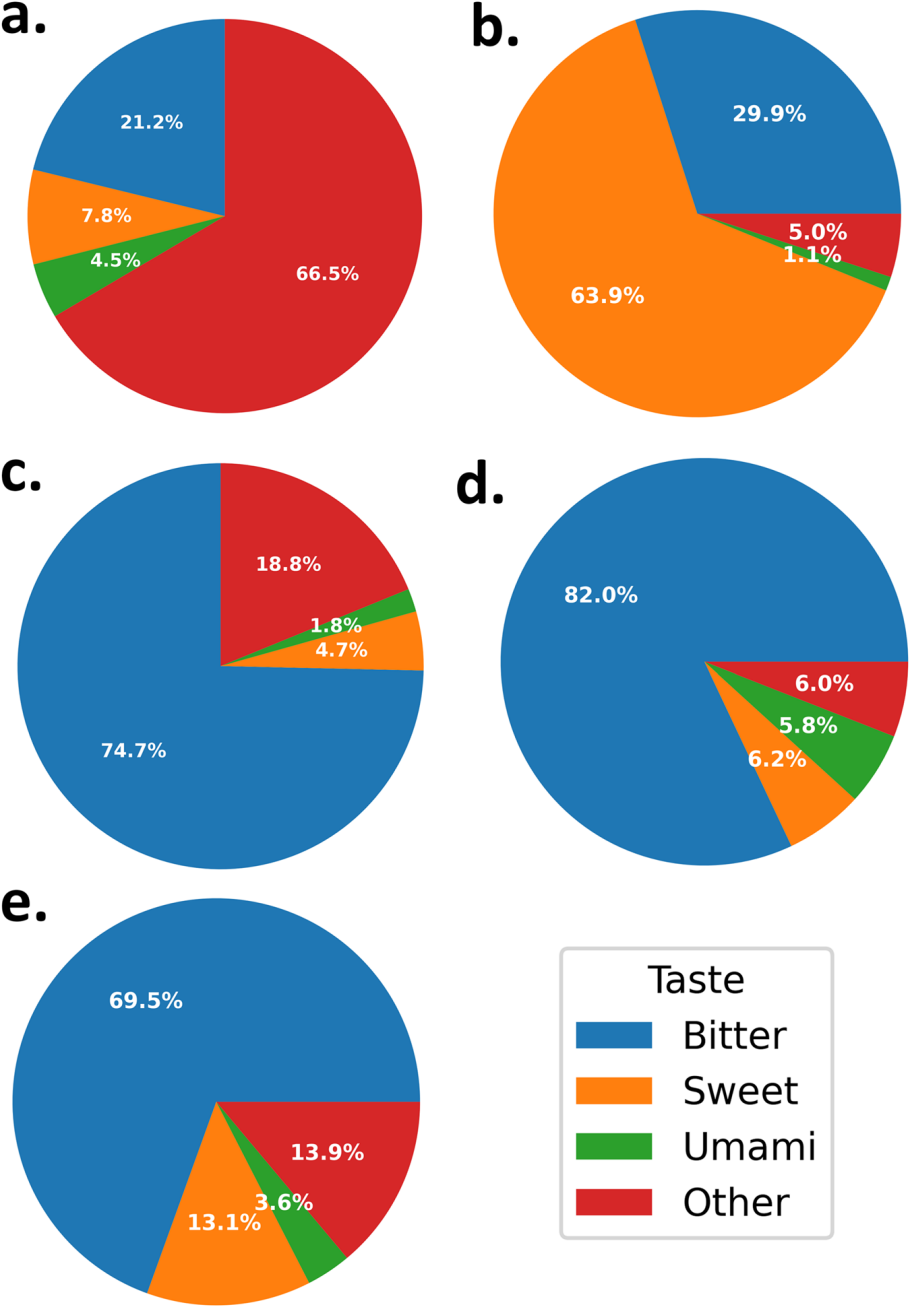
Distribution of predicted tastes for external databases. The pie charts represent the distributions of bitter, sweet umami and other tastes in the analysed external datasets, including (**a**) FooDB, (**b**) FlavorDB, (**c**) PhenolExplorer, (**d**) Natural Product Atlas, and (**e**) PhytoHub.

The distributions of predicted tastes for each of the external datasets are represented in Fig. 4 (see also Supplementary Table 2 for detailed numbers).

### VirtuousMultiTaste platform

The multi**-**taste predictor that was developed has been integrated into a web-based interface (https://virtuous.isi.gr/#/virtuous-multitaste). This platform serves as a user-friendly graphical interface for conducting analyses on chemical compounds expressed in different notations such as SMILES, FASTA, InChI, SMARTS, or PubChem compound names. When the user provides a PubChem name, the system searches the PubChem database for the corresponding compound and retrieves its SMILES representation to perform the multi-taste prediction. The platform design follows the separation of concerns principle between the presentation layer (front-end) and the data access layer (back-end). The front end is the visible part of the application that users interact with on their devices. It allows users to input compounds directly into a text field or upload a file that consists of several compounds, each one written on a separate line. Furthermore, the users may choose to specify the annotation format of their inputs or let the system recognize it automatically. Once the analysis is completed, the results are displayed in a table format, showing the queried compounds’ SMILES, their 2D molecular representation, and the taste prediction result presented both in a textual format and as a spider chart. Moreover, next to each compound there are two buttons for downloading the calculated molecular descriptors or the best 15 descriptors on which the final model relies. The front-end development utilizes the Ionic framework, chosen for its wide range of user interface components suitable for both browsers and mobile devices. The back-end component is a cloud-based web service implemented using the Flask micro-framework, known for its lightweight yet robust nature. It receives input from the front end, executes the VirtuousMultiTaste analyser, and sends the results back to the front end. To facilitate this information exchange, a RESTful API is provided, which accepts and transmits data in the form of JavaScript Object Notation (JSON).

## Discussion

Machine learning techniques have demonstrated their pivotal role in advancing the development of prediction tools and digital support systems across diverse fields, such as nutrition and agri-food research^46–52^. In our previous works related to the prediction of taste, a bitter/sweet predictor, named VirtuousBitterSweet^27^, and an umami predictor, named VirtuousUmami^30^, were created. Herein, we developed a machine learning-driven taste predictor capable of identifying bitter, sweet, and umami from other taste sensations starting from the molecular structures of a query compound.

The data used to train the model was obtained from a variety of previously defined databases, including samples for each of the taste sensations under investigation. Two-dimensional Mordred molecular descriptors were used to extract features from the data. It is noteworthy that previous studies have achieved notable outcomes in taste prediction within the domain of 2D molecular descriptors alone^22,25,27,30^. This advancement marks a significant stride, as 2D molecular descriptors are computationally less demanding and remain unaffected by variations in three-dimensional molecular structures compared to 3D molecular descriptors. The extremely high number of molecular features (1613), especially in comparison to the number of compounds in the training dataset, was reduced using the Mann-Whitney statistical analysis, pinpointing 1307 statistically significant descriptors. The PCA analysis showed that statistically significant features can discriminate remarkably better between the four different tastes if compared to the analysis considering all the descriptors (see also Supplementary Fig. 1). The number of features used for the taste prediction has been further refined during the model development: the employed heuristic multi-objective Pareto-based evolutionary optimization algorithm was able to select the Random Forest (RF) as the most appropriate classifier, to choose the optimal parameters being 95 Decision Trees and the most important and informative features on which the model relies. Ultimately, we ended up with only 15 features: this allows us to not only achieve better performance but also simplify the model and improve its explainability. Interestingly, the most frequent descriptor class among the 15 selected features utilizes the so-called Autocorrelation of a Topological Structure (ATS) which quantifies the spatial arrangement and distribution patterns of atoms or molecular fragments within a molecule. It calculates the correlation between the properties of a specific atom or fragment and the properties of other atoms or fragments within a defined distance in the molecule. This approach provides information about the local environment and structural features of a molecule. In particular, the autocorrelation descriptors were computed using the Moreau-Broto autocorrelation weighted by Sanderson electronegativity (ATSC0se, AATSC2se, ATSC1se), by mass (ATSC1m and AATSC0m), by Gasteiger charge (ATSC0c, ATSC1c), by atomic number (ATSC1Z, AATSC1Z), by ionization potential (AATS0i), by polarizability (ATSC1p), by Allred-Rocow electronegativity (AATSC2are), by Pauling electronegativity (AATSC1pe) and by intrinsic state (ATSC4s). Interestingly, the selected features are mostly associated with charge distribution, electronegativity, and polarizability, which are fundamental characteristics for the effective interaction of tastants with taste receptors capable of recognizing sweet, bitter, and umami tastes. Although there has been a significant reduction in the number of features, it remains challenging to intuitively elucidate the chemical and physical properties of tastants solely based on the 15 most important features. To enhance the model’s explainability, future studies should prioritize the use of simpler descriptors or the development of specific methodologies to intuitively correlate the molecular descriptors to the relative structural features or functional groups. This will enable a clearer understanding of the underlying factors contributing to the prediction of multiple taste sensations.

The proposed machine learning model was benchmarked against commonly used machine learning methods and pipelines. RFs, SVMs and XGBoost were applied to the same dataset tuning their parameters with grid search and selecting their features using the minimum Redundancy Maximum Relevance (mRMR) method (see also Supplementary Table 3). VirtuousMultiTaste outperforms the other three classifiers (RF, XGBoost and SVM) across all performance metrics. Moreover, VirtuousMultiTaste has been compared with previous tools dedicated to the prediction of only the bitter and sweet taste, i.e., VirtuousSweetBitter^27^ and BitterSweet^25^. These two tools were selected because they were the only ones readily accessible and usable for a proper performance assessment. To have a fair comparison, we selected compounds not included in any of the training sets of the three taste predictors, resulting in a final external test of 869 compounds (409 bitter and 460 sweet). We evaluated the performance for predicting separately the bitter and the sweet taste, thus accessing the ability of each classifier to effectively detect bitter/non-bitter (Supplementary Table 4) or sweet/non-sweet (Supplementary Table 5) molecules. VirtuousMultiTaste exhibited slightly superior performance in bitter taste prediction compared to the other tools, with all performance metrics (ACC, F1, F2, Precision, and Recall) hovering around 83%. In comparison, VirtuousSweetBitter and BitterSweet achieved performance levels of approximately 80% and 77% across all metrics, respectively (Supplementary Table 4). Regarding the prediction of sweet taste, VirtuousMultiTaste performed at intermediate values between VirtuousSweetBitter and BitterSweet. However, VirtuousMultiTaste maintained a satisfactory level of performance and showed commendable predictive capabilities (Supplementary Table 5). As for the comparison with tools for umami taste prediction, we were limited to comparing VirtuousMultiTaste with our previous tool, VirtuousUmami^30^, as all other previously developed predictors, such as iUmami-SCM^28^ and UMPred-FRL^29^, are based on the peptide sequence of the compound and therefore cannot be applied to more general molecular annotations, such as SMILES. It was not possible to create an independent test set for both VirtuousMultiTaste and VirtuousUmami, due to the limited number of proven umami compounds in the literature. Comparing the performance metrics in the relative cross-validation sets, VirtuousMultiTaste and VirtuousUmami achieved similar accuracy (around 96%) and AUC scores (above 96%) in the cross-validation set, whereas slightly lower values for F1, F2, Precision and Recall was achieved by the present tool (see also Supplementary Table 6). Moreover, testing the two tools with non-umami compounds not used for training, including sweet, bitter, and other compounds, VirtuousMultiTaste and VirtuousUmami both achieved an accuracy of over 99%. Certainly, it is important to note that this comparison only allows us to consider the accuracy in predicting the negative class, i.e., non-umami, and therefore it represents a partial comparison. It is also worth mentioning that the performance values for the umami taste (Supplementary Table 6) generally surpass those for bitter and sweet tastes (Supplementary Table 4 and Supplementary Table 5). This can be attributed to two main factors: (i) the limited number of confirmed umami compounds in the literature led to obtaining umami comparison metrics on the relative cross-validation set of the two compared tools, not on a dataset excluded from the respective training sets, as done for bitter/sweet comparison; (ii) the chemical domain of experimentally-known umami compounds is notably narrower than those for sweet and bitter chemicals, simplifying the model’s training and resulting in better umami taste performance Furthermore, we tested three recently proven umami peptides, i.e., FLNQDEEAR (FR-9), FNKEE (FE-5), and EEFLK (EK-5)^53^, and all of them have been predicted as umami by VirtuousMultiTaste. Finally, the presence of 90 non-peptides umami compounds coming from the ChemTastesDB in the training set enabled the development of a tool capable of predicting umami compounds not necessarily peptides, allowing the exploration of a wider chemical space compared to previous umami predictors^28–30^. In summary, VirtuousMultiTaste has demonstrated remarkable performance compared to previous taste-specific predictors, with the added advantage of being able to predict multiple taste sensations at the same time.

Regarding the range of applicability for the proposed model, we conducted an evaluation to assess whether the model’s performance exhibited variations based on the similarity between the tested compounds and the chemicals used during the training phase. Accordingly to our previous works^27,30^, we evaluated the average similarity score between test and training compounds: (i) the Morgan Fingerprints (1024 bits, radius 2) were calculated using RDKit for all the compounds in the dataset; (ii) the Tanimoto similarity index was computed between each molecule in the test set and the previously-defined fingerprints; (iii) then the average similarity score was calculated by averaging the similarity scores of the 5 most similar couple of compounds. Compounds in the test set have been divided into 10 quartiles according to the average similarity score and the model performance has been evaluated on each quartile separately (see also Supplementary Fig. 4). Since the performance remained remarkably stable for each similarity quartile, we concluded that the model was able to preserve its performance regardless of the similarity of the query compounds with the ones in the training set, thus ensuring a general applicability domain. This fact can also be attributed to the composition of the training database used for the model, which encompasses three of the five fundamental taste sensations and includes a fourth category, i.e. “other taste”, including a wide spectrum of compounds with distinct tastes. As a result, the curated dataset might embrace a substantial portion of the chemical space that underlies the taste sensations of tastants. It is important to underscore that a primary limitation of the current study is the relatively limited number of umami compounds in the training dataset, particularly in comparison to the number of compounds representing other taste sensations. As previously noted in existing literature^28–30^, augmenting the number of experimentally identified umami samples would not only enhance the predictive performance of the algorithm but also bolster its robustness.

VirtuousMultiTaste predictor also demonstrates its versatility by accommodating various types of molecular structure notations, such as SMILES, FASTA, InChI, SMARTS, or PubChem name. This capability enables the screening of diverse compound types, thereby creating opportunities to explore extensive molecular databases for the identification of tastes. In this context, we applied the VirtuousUmami predictor to five distinct external databases associated with food or natural compounds, namely FooDB, FlavorDB, PhenolExplorer, Natural Product Atlas, and PhytoHub, to pinpoint the distributions of tastes. The representation of umami taste is relatively lower compared to sweet or bitter tastes across the screened databases. This outcome aligns reasonably with expectations, as umami taste is closely associated with the protein content of food, which, in turn, is underrepresented within the selected external databases and in general if compared to sweet or bitter. In contrast, the bitter taste predominates in the PhenolExplorer, Natural Product Atlas, and PhytoHub databases. This finding aligns with the outcomes of previous machine learning-based classifiers, indicating that a remarkable proportion of natural compounds exhibit a bitter taste^24,25,54^. Concerning FlavorDB, a substantial majority of the molecules were projected to possess sweet (63.9%) or bitter (29.9%) characteristics. It is noteworthy that the screening of FlavorDB yielded a distribution similar to that reported in previous literature, albeit with a considerably larger number of processed compounds (approximately 400 more)^25^.

As a further example of applicability, this platform can be used also for food screening, enabling the analysis of the chemical composition of desired foods. Therefore, we examined coffee and chocolate using their relative composition as reported in FooDB. The taste analysis was conducted directly from our platform in the Foods tab (https://virtuous.isi.gr/#/foods), where we provided an interactive exploration of the FooDB and enabled the direct execution of the multi-taste prediction for each of the compounds present in the investigated foods. Regarding the screening of coffee, 130 compounds were predicted as bitter, 44 as sweet, 4 as umami and 14 as other tastes, thus pointing out that coffee is characterized by mainly bitter compounds. Chocolate also presented a predominance of bitter compounds, with a taste profile consisting of 96 bitter compounds, 33 sweet compounds, 4 umami compounds and 13 other taste compounds. Both food screenings seem in accordance with the primary taste perceptions for these foods, which are indeed bitter. However, it is crucial to mention that this model can only predict the taste of individual chemical compounds and lacks the capability to predict the overall taste of food, which is influenced by various factors such as the specific concentrations of its ingredients, the food processing techniques (e.g. baking, frying, dehydration, steam cooking, among others), visual aspect (colour, shape, dimension), touch, taste, and odour. This type of feature could be employed in future holistic models able to consider not only the chemical structures of food ingredients but also other crucial factors underlying the overall taste.

The creation of a user-friendly web interface (accessible at https://virtuous.isi.gr/#/virtuous-multitaste) was driven by the objective of ensuring the accessibility of the multi-taste prediction model to users who may lack experience or familiarity with technical coding. Additionally, the web interface is complemented by a corresponding GitHub repository (https://github.com/lorenzopallante/VirtuousMultiTaste) that provides access to the technical Python codes for those interested in further exploration and customization.

In conclusion, a machine learning-based taste predictor, named VirtuousMultiTaste, was developed for identifying bitter, sweet, and umami from other taste sensations by combining heuristic optimization and nonlinear machine learning methods. VirtuousMultiTaste represents a paramount tool for rapidly screening compound databases to identify a diverse array of potential candidate compounds with anticipated taste properties. In a broader sense, it is worth mentioning that the future perspectives beyond the present work also include the possibility of effectively predicting the remaining two taste sensations, i.e., sour and salty, to obtain a singular and comprehensive taste predictor capable of predicting all five fundamental tastes at once. This research lays the foundations for future works aimed at developing specific models capable of predicting the sensory profile of foods and engineering new products with desired tastes or properties, potentially impacting various fields, such as nutrition, precision medicine, the food market, and beyond.

## Methods

### Database and data curation

The starting dataset was built by gathering publicly available datasets reporting chemicals with verified taste and divided into nine taste classes (sweet, bitter, non-sweet, umami, tasteless, sour, salty, multitaste and other tastes). In detail, we utilized the sweet/bitter database curated for our previous VirtuousSweetBitter classifier^27^, umami and non-umami samples from the UMP442 database^28^ used for our VirtuousUmami classifier^30^, as well as, compounds with miscellaneous tastes from the ChemTastesDB^55^. A summary of the collected compounds from these taste databases is reported in the Supplementary Information (Supplementary Table 7). After removing compounds labelled as Multitaste, since they were not enough to justify a multi-labelling approach, we used only three classes (sweet, bitter and umami) and all other classes were considered as Other because of relatively low sample size. In this context, it is important to note that sour and salty tastes have not been treated as specific taste classes due to the scarcity of retrieved data, i.e. 38 and 12 compounds, respectively. Therefore, this work is focused on the prediction of four classes i.e., Sweet, Bitter, Umami and Other.

Regarding the Bitter and Sweet compounds, a total of 5290 compounds (2741 sweet and 2549 bitter) with their SMILES description were used as the initial database. All the SMILES were checked using the RDKit library^56^, searching for the relative correct SMILES in the PubChem database (if necessary) and removing duplicates, as well as compounds with incorrect SMILES.

Umami compounds were gathered from the UMP442 dataset and the ChemTastesDB. The UMP442 dataset collects 140 umami molecules from previous literature^57,58^ and the BIOPEP-UWM database^59^, whereas the ChemTastesDB contains 98 umami compounds. These peptides were collected using their amino acid sequences and converted into their SMILES representation using the RDKit package, as done in previous work^30^.

Concerning the samples categorized under the “Other” class, which includes compounds not falling into the bitter, sweet, or umami categories, a total of 208 tasteless compounds were gathered from previous literature^60–62^, and an additional 203 tasteless compounds were sourced from ChemTasteDB. Moreover, 370 compounds with other taste sensations from ChemTasteDB were included. Consequently, the dataset comprises 781 compounds labelled as “Other.”

In summary, our initial database was composed of 6309 compounds - 2741 sweet, 2549 bitter, 238 umami, and 781 other - all with their SMILES representation.

Following pre-processing protocols used in previous literature^25,27,30^, all SMILES were processed with the ChEMBL Structure Pipeline^63^. This step is aimed at identifying and addressing any potential issues in the molecular structures, as well as standardising the SMILES representation across the entire dataset. In detail, the protocol runs a molecule checker on the compound structure, standardizes chemical structures and generates the parent molecule representation based on a set of predefined rules. Incorrect SMILES and duplicates were then removed, obtaining a final dataset of 4717 compounds (1904 sweet, 1937 bitter, 227 umami and 649 other). A schematic summary of the refined database is reported in Supplementary Table 8.

To train the model, a random subset was then extracted, selecting 360 sweet, 360 bitter, 227 umami and 360 other chemicals. Moreover, the umami class was oversampled with additional 133 umami samples to match the size of the other classes using the Adaptive Boosting (AdaBoost) algorithm, which will be described in detail in the *Model construction and performance evaluation* section. The remaining 1544 sweet, 1577 bitter and 289 other peptides were used for external testing to examine the generalization properties of the trained models. A table summarising the division between the training and the test set is reported in the Supplementary Information (Supplementary Table 9).

### Molecular descriptors and dimensionality reduction

Following our previous works^27,30^, the molecular features calculation was achieved using the 1613 2D Mordred descriptors^64^ for each compound. The comprehensive compilation of employed Mordred descriptors can be accessed at https://mordred-descriptor.github.io/documentation/master/descriptors.html. The 2D Mordred descriptors furnish valuable insights into compounds, encompassing essential molecular information such as molecular weight, the count of distinct atom types, bond types, hybridization degree, spectral diameter, detour index, count of hydrogen donors and acceptors, molecular distance edge between distinct atom types, the polarizability of atoms and bonds, as well as the topological polar surface. Furthermore, these descriptors encompass additional features derived from symbolic representations, including the Zagreb index, adjacency matrix descriptors, Moreau–Mroto descriptors, Moran coefficients, Geary coefficients, and descriptors delineating the Burden matrix and Barysz matrix^64^. Furthermore, the dataset underwent pre-processing steps to prepare it for input into the ML model. Features with a high percentage of missing values (>30%) were discarded, while the remaining missing values were imputed using the kNN-impute method with *k* = 20^65^. Subsequently, the data were arithmetically normalized in the range of [0-1]. Data pre-processing, statistical analysis, and the generation of additional plots, such as ROC curves and bean plots, were performed using the InSyBio Biomarkers Tool^66^.

### Model construction and performance evaluation

Based on our previous work^30^, we used a hybrid approach of heuristic optimization and nonlinear machine learning to develop classification models. Specifically, we used an ensemble dimensionality reduction technique that employed a heuristic multi-objective Pareto-based evolutionary optimization algorithm to (a) identify the optimal feature subset to use as input to the classifiers, (b) select the most appropriate classifier among SVM and RF, and (c) select the optimal parameters for the classifier (e.g., C and gamma for SVM, the number of trees for RF). By utilizing the multi-objective Pareto-based approach, we sought to optimize prediction performance, minimize the selected features, and simplify the classification model. The weights used for the optimization objectives were Selected Features Number Minimization 1, Accuracy (ACC) 10, F1 score 10, F2 score 1, Precision (PRC) 1, Recall (REC) 10, ROC-AUC (AUC) 1, Number of SVs or Trees Minimization 1, and Manhattan Distance 1. These weights allowed us to effectively address the imbalanced nature of our classification problem. To get a better handle on the unbalanced nature of the multi-class taste prediction problem and improve the accuracy of the prediction models, we additionally used the Adaptive Boosting (AdaBoost) algorithm as an additional pre-processing step before the training^67^. This algorithm performs boosting, assigning weights over the internal training set in the cross-validation iterations. This algorithm assigns higher weights to the minority class and then generates synthetic copies of the minority class samples until all the classes are balanced. This is especially helpful for the unbalanced class, i.e., the umami one. The outcome is the generation of multiple models that exhibit similar performance concerning the user-defined objectives, which correspond to the Pareto set of optimal solutions.

The evolutionary algorithm was applied to a population of 100 individuals, and the termination criterion was set to a maximum of 200 generations. Ten different runs were conducted to deal with the stochastic nature of the algorithm, and the results presented are the average performance of these runs. Convergence of the algorithm (average performance differing less than 5% from that of the best-performing individual) was found after 50 generations for each independent run. This finding affirmed that the selected maximum number of generations was appropriate for this problem. Additional parameters of the evolutionary algorithm were set to their default values as suggested by the InSyBio Biomarkers tool user manual (arithmetic crossover probability: 0, mutation probability: 0.01, two-point crossover probability: 0.9). Stratified 10-fold cross-validation was used to train and test the prediction models. Further details on the implementation of the trained models and a summary of the performance metrics used are available in the Supplementary Information.

**Fig. 5:**
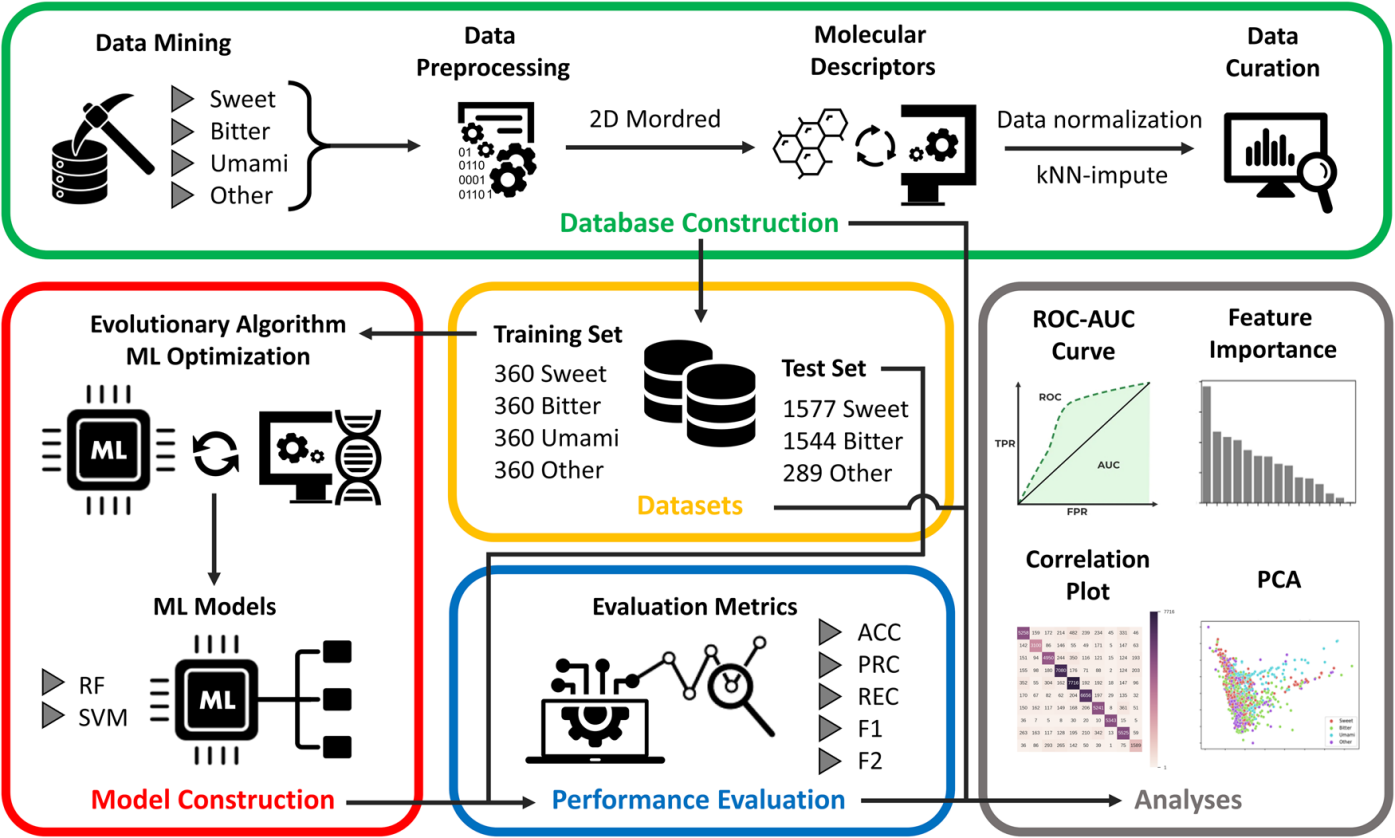
Flowchart of VirtuousMultiTaste. The flowchart represents the major stages in the workflow of the proposed taste prediction tool.

The pipeline of the adopted methods is presented in Fig. 5.

### Model explainability and applicability

Model explainability was performed using SHAP (SHapley Additive exPlanations)^68^. SHAP values offer insights into the contribution of individual features to the model’s final prediction, facilitating the interpretation of its decision-making process. To compute the SHAP values, we employ the TreeExplainer method to generate an explainer object for the Random Forest (RF) model, since RFs were selected over SVMS from the multi-objective optimization framework deployed. The explainer object enables the computation of the SHAP values for each feature in the dataset. Considering the nature of the classification problem, we obtain the SHAP values for each class, providing insight into the contribution of each feature to the probability of that class. The SHAP values analysis of each taste reveals the features with the highest importance for each taste and their contribution to the final prediction.

The developed model was also tested on external databases related to foods or natural products, including FooDB,(https://foodb.ca/), FlavorDB (https://cosylab.iiitd.edu.in/flavordb/), PhenolExplorer (http://phenol-explorer.eu), Natural Product Atlas (https://www.npatlas.org/), and PhytoHub (https://phytohub.eu/). Coherently with the pre-processing of the dataset used for training and testing, each external database was first checked for missing SMILES or data, standardised with the ChEMBL Structure Pipeline, and featurized using Mordred descriptors.

### Supplementary information

Supplementary information

## Data Availability

All data needed to replicate the present work are available at https://github.com/lorenzopallante/VirtuousMultiTaste.

## Code availability

A public GitHub repository is available at https://github.com/lorenzopallante/VirtuousMultiTaste containing all source code and scripts used for the present work.
