# Supplementary material for: Predicting multiple taste sensations with a multiobjective machine learning method
Source: NPJ Sci Food. 2024 Jul 25;8:47. doi: 10.1038/s41538-024-00287-6 (PMC11272927; doi:10.1038/s41538-024-00287-6)
Supplement: Supplementary file 1 — Supplementary information [file 41538_2024_287_MOESM1_ESM.pdf]

Supplementary Information for manuscript *"Predicting Multiple Taste Sensations with a Multiobjective Machine Learning Method"*

Lampros Androutsos[1†], Lorenzo Pallante[2†], Agorakis Bompotas[3], Filip Stojceski[4], Gianvito Grasso[4], Dario Piga[4], Giacomo Di Benedetto[5], Christos Alexakos[3], Athanasios Kalogeras[3], Konstantinos Theofilatos[1*], Marco A. Deriu[2] and Seferina Mavroudi[1,6]

[1] InSyBio PC, Patras, 265 04, Greece

[2] Polito[BIO]MedLab, Department of Mechanical and Aerospace Engineering, Politecnico di Torino, Torino, 10129, Italy

[3] Industrial Systems Institute, Athena Research Center, 265 04 Patras, Greece.

[4] Department of Innovative Technologies, Dalle Molle Institute for Artificial Intelligence, Lugano-Viganello, 6962, Switzerland

[5] Enginlife Engineering Solutions, Turin, Italy

[6] Department of Nursing, University of Patras, 265 04 Patras, Greece.

* k.theofilatos@insybio.com

† Joint First Authors

Results – Model Performance

The following evaluation functions were employed to guide the optimization process, aiming to maximize predictive performance, minimize the number of selected features, and prioritize the simplicity of the classification model:

- Selected Features Number Minimization (SFNM):

$$SFNM = \frac{1}{1 + Number\ of\ selected\ features}$$

- Accuracy (ACC):

$$ACC = \frac{Tp + Tn}{Tp + Fp + Tn + Fn}$$

  where Tp represents the true positives, Tn the true negatives, Fp the false positives and Fn the false negatives.

- Precision (PRC):

$$PRC = \frac{Tp}{Tp + Fp}$$

- Recall (REC):

$$REC = \frac{Tp}{Tp + Fn}$$

- F1 Score (F1):

$$F1 = \frac{2 * \mathrm{PRC} * \mathrm{REC}}{\mathrm{PRC}\ + \mathrm{REC}}$$

- F2 Score (F2):

$$F2 = \frac{5 * \mathrm{PRC} * \mathrm{REC}}{4 * \mathrm{PRC} + \mathrm{REC}}$$

- ROC-AUC: Area Under the Receiver Operating Characteristic curve of Sensitivity/Specificity

- Number of SVs or Trees Minimization: Number of Samples in Training Set/Number of Support Vectors of the trained Support Vector Regression Problem

Supplementary Table 1 | Performance of the 20 developed RandomForest (RF) models.

Model	*ACC*	*F1*	*F2*	*Precision*	*Recall*	*AUC*	*#Features*
**1**	71.96%	74.20%	73.19%	78.53%	71.96%	0.87	191
**2**	**71.96%**	**74.32%**	**73.10%**	**78.98%**	**71.76%**	**0.87**	**15**
**3**	72.73%	74.88%	73.92%	79.08%	72.73%	0.88	183
**4**	73.08%	75.06%	74.18%	78.96%	73.08%	0.88	191
**5**	72.52%	74.64%	73.69%	78.78%	72.52%	0.88	190
**6**	72.43%	74.56%	73.61%	78.70%	72.43%	0.88	198
**7**	73.17%	75.17%	74.27%	79.03%	73.17%	0.88	180
**8**	69.50%	71.77%	70.71%	75.98%	69.50%	0.84	199
**9**	72.46%	74.58%	73.63%	78.72%	72.46%	0.88	194
**10**	73.02%	74.96%	74.11%	78.83%	73.02%	0.88	192
**11**	73.26%	75.23%	74.35%	79.08%	73.26%	0.88	186

**12**	72.87%	74.99%	74.04%	79.10%	72.87%	0.88	191
**13**	72.14%	74.40%	73.39%	78.82%	72.14%	0.88	187
**14**	73.20%	75.12%	74.27%	78.92%	73.20%	0.87	186
**15**	72.64%	74.61%	73.72%	78.36%	72.64%	0.88	188
**16**	73.31%	75.32%	74.43%	79.24%	73.31%	0.88	191
**17**	72.52%	74.71%	73.71%	78.87%	72.52%	0.88	192
**18**	72.38%	74.49%	73.55%	78.66%	72.38%	0.88	185
**19**	73.31%	75.28%	74.41%	79.16%	73.31%	0.88	200
20	72.93%	74.95%	74.05%	78.89%	72.93%	0.88	175

Results – Dimensionality Reduction

The Principal Component Analysis (PCA) was utilized to illustrate the challenge of distinguishing between the four taste classes using linear dimensionality reduction techniques. In the PCA on the complete set of features, the first principal component (PC0) explains 73.31% of the variance, the second (PC1) explains 6.64%, and the third (PC2) explains 2.09%. Conversely, in the PCA for the statistically significant features, PC0 accounts for 42.15% of the variance, PC1 for 10.61%, and PC2 for 6.48%. Considering the overall explained variance, it was decided to confine the PCA analysis to the first three principal components. Introducing a fourth principal component would have added an extra 1.91% and 4.35% to the total variance for the dataset with all features and the one containing only statistically significant features, respectively.

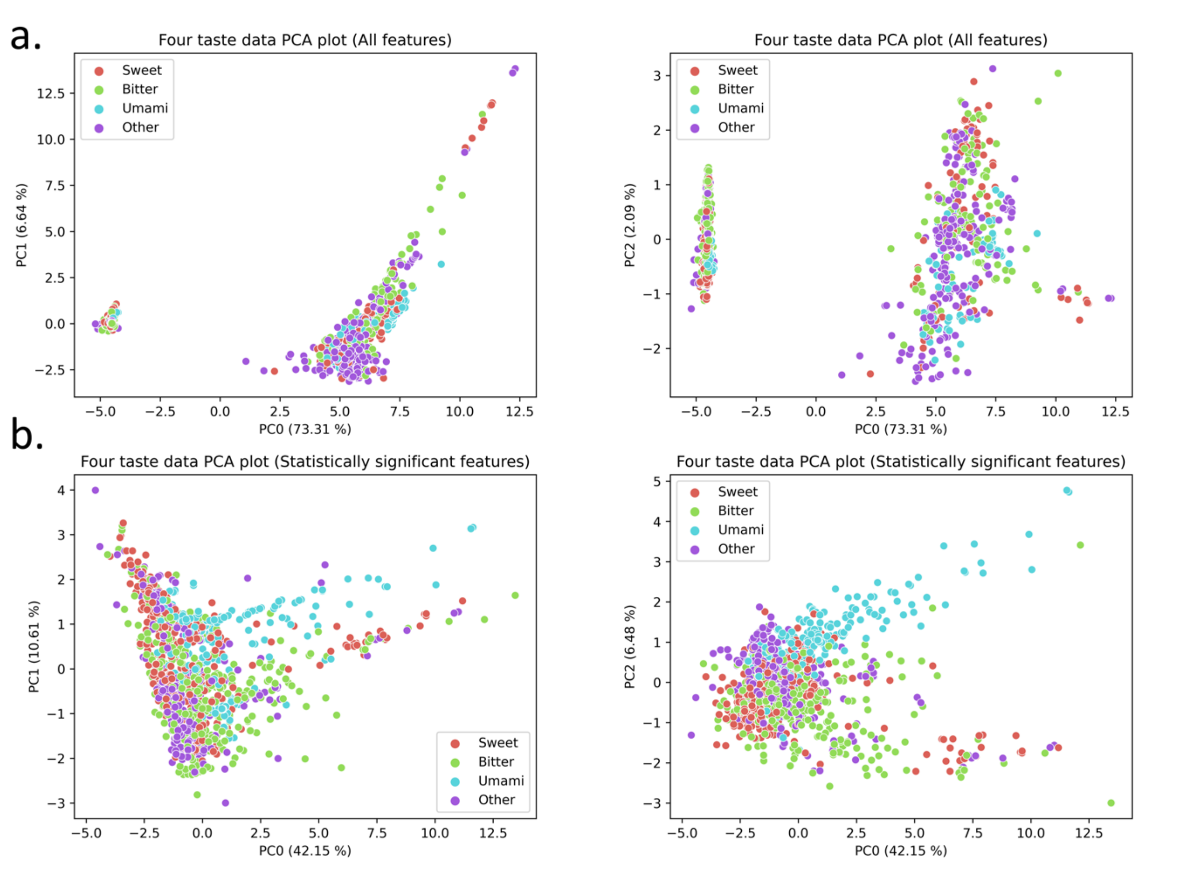

Supplementary Figure 1 | Principal Component Analysis (PCA). PCA was performed using (a) all features and (b) 1306 statistically significant differentiated features derived from the training process and the first three principal components are represented.

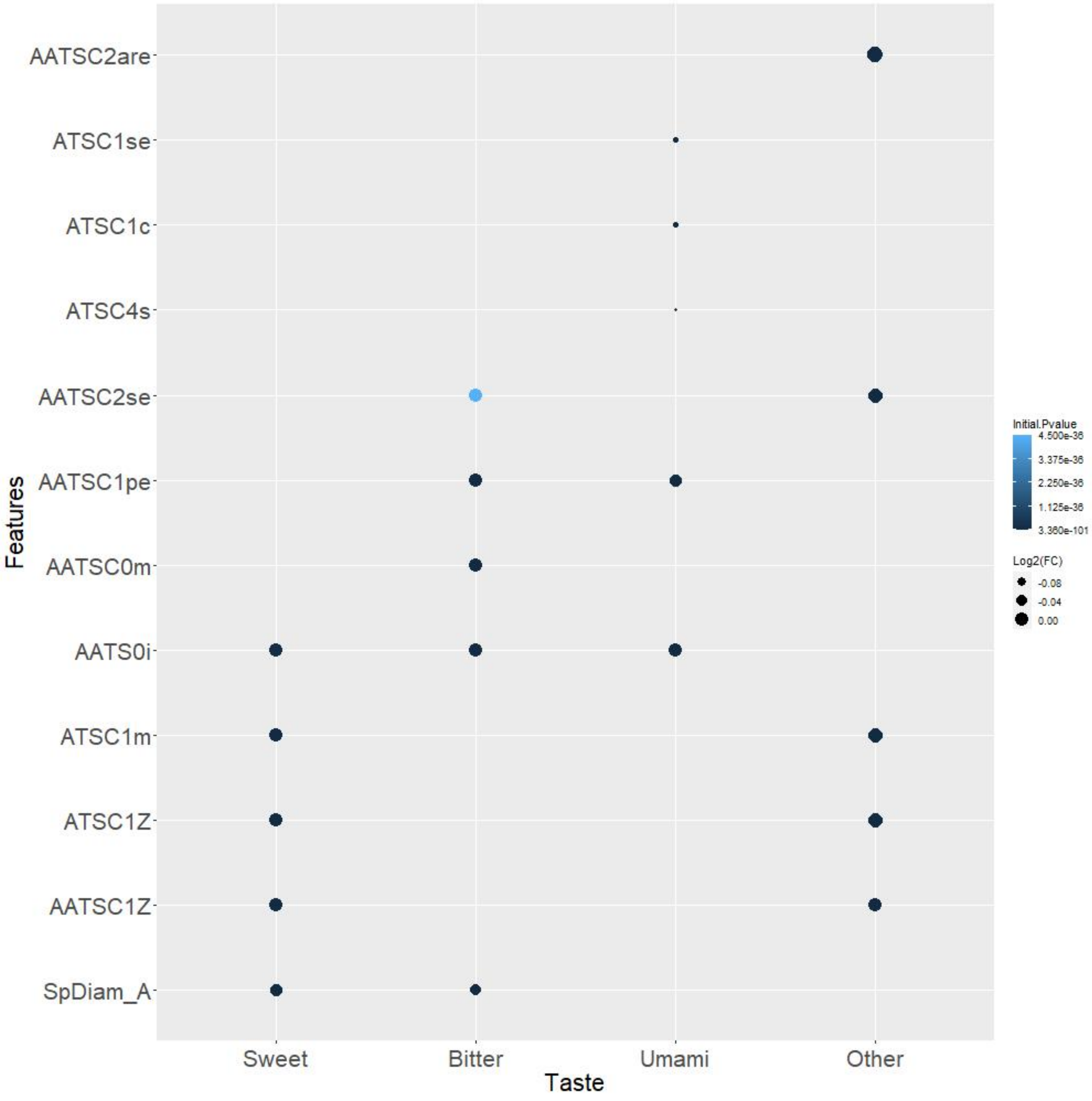

Supplementary Figure 2 | Heatmap of the top 5 features differentiating each taste from the rest in the training dataset.

Results – Feature Importance

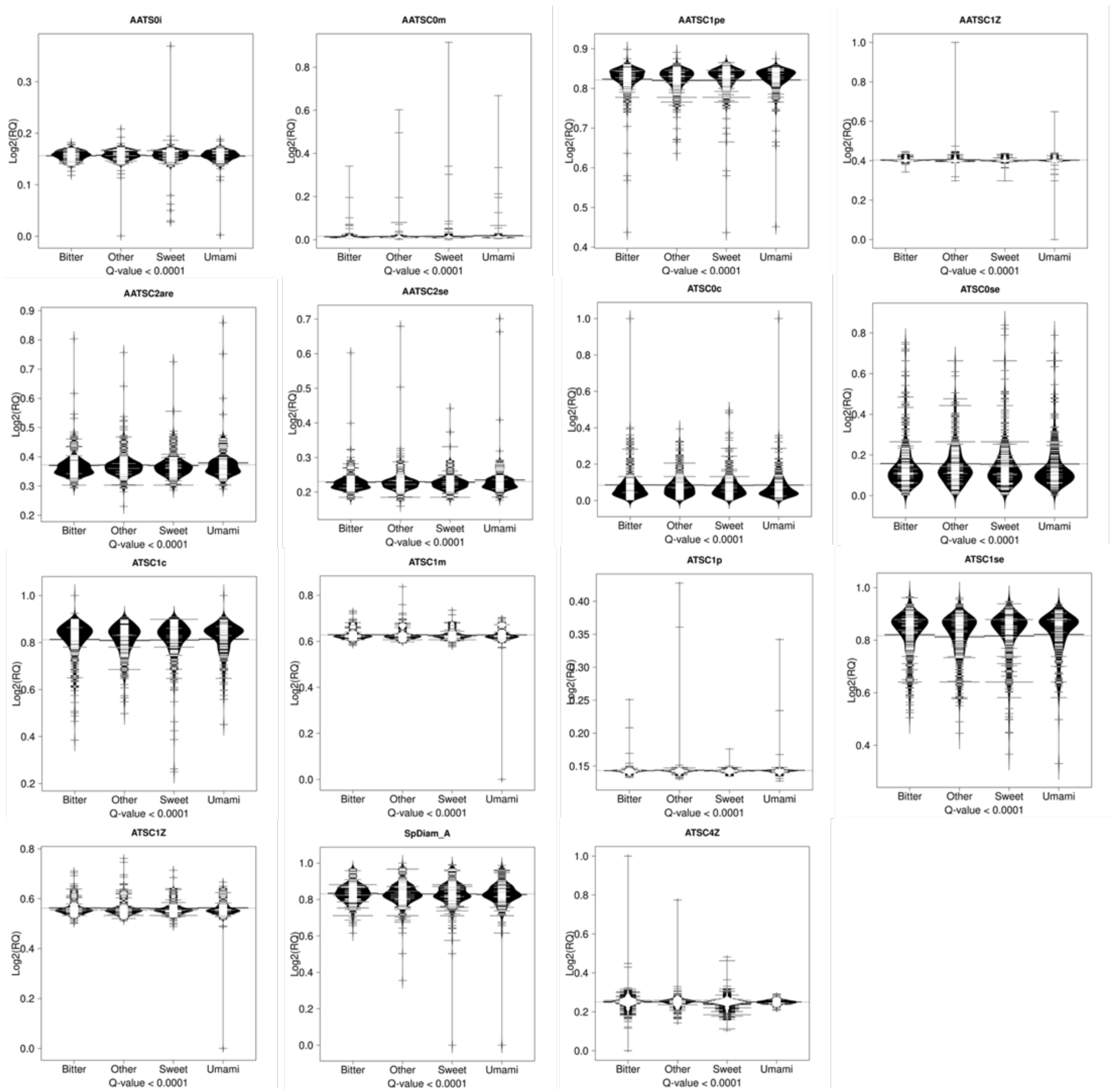

Supplementary Figure 3 | Violin plots for the 15 most significant features on which the prediction relies.

Results – External datasets

Supplementary Table 2 | Predictions on external datasets with VirtuousMultiTaste

	Total	Sweet	Bitter	Umami	Other
**FooDB**	69309	5375 (7.8%)	14693 (21.2%)	3149 (4.5%)	46092 (66.5%)
**FlavorDB**	2599	1661 (63.9%)	778 (29.9%)	29 (1.1%)	131 (5.0%)
**PhenolExplorer**	489	23 (4.7%)	365 (74.7%)	9 (1.8%)	92 (18.8%)
**Natural Product Atlas**	32491	2019 (6.2%)	26653 (82.0%)	1880 (5.8%)	1939 (6.0%)
**PhytoHub**	1746	228 (13.1%)	1213 (69.5%)	62 (3.6%)	243 (13.9%)

Discussion

Supplementary Table 3 | Comparison of performance using the proposed pipeline versus RF, SVM and XGBoost classifiers using the mRmR for feature selection.

	ACC	*F1*	*F2*	*Precision*	*Recall*	*AUC*
VirtuousMultiTaste	71.96%	74.32%	73.10%	78.98%	71.76%	0.87
RF (mRmR)	61.22%	65.31%	63.17%	72.44%	61.22%	0.75
XGBoost (mRmR)	61.54%	65.39%	63.48%	72.66%	61.54%	0.77
SVM (mRmR)	44.12%	45.15%	47.11%	70.55%	44.12%	0.65

Supplementary Table 4 | Comparison between VirtuousMultiTaste, VirtuousSweetBitter and BitterSweet regarding the prediction of the bitter taste. The comparison was performed by running the taste predictions on compounds in the test set of the VirtuousMultiTaste tool and not present in the training set of the VirtuousSweetBitter and BitterSweet models.

	ACC	*F1*	*F2*	*Precision*	*Recall*
VirtuousMultiTaste (Bitter)	83.43 %	83.60 %	83.45 %	83.42 %	83.41 %
VirtuousSweetBitter (Bitter)	80.02 %	81.02 %	80.47 %	79.98 %	80.09 %
BitterSweet* (Bitter)	77.78 %	77.92 %	77.83 %	77.77 %	77.78 %

*one compound was not predicted by the BitterSweet model.

Supplementary Table 5 | Comparison between VirtuousMultiTaste, VirtuousSweetBitter and BitterSweet regarding the prediction of sweet taste. The comparison was performed by running the taste predictions on compounds in the test set of the VirtuousMultiTaste tool and not present in the training set of the VirtuousSweetBitter and BitterSweet models.

	ACC	*F1*	*F2*	*Precision*	*Recall*
VirtuousMultiTaste (Sweet)	72.50 %	75.56 %	73.44 %	72.11 %	72.37 %
VirtuousSweetBitter (Sweet)	79.17 %	80.36 %	79.73 %	79.12 %	79.27 %
BitterSweet* (Sweet)	69.93 %	72.01 %	70.72 %	69.65 %	69.90 %

*one compound was not predicted by the BitterSweet model.

Supplementary Table 6 | Comparison between VirtuousMultiTaste and VirtuousUmami on their relative cross-validation sets. Note that the performance is related to a different dataset since no independent test set can be created for both tools due to the limited number of umami compounds available in the literature.

	ACC	*F1*	*F2*	*Precision*	*Recall*	*AUC*
VirtuousMultiTaste	95.99%±0.16	88.49%±0.51	88.64%±0.68	87.61%±0.67	87.40%±1.17	0.98±0.005
VirtuousUmami	95.86%±1.89	96.70%±2.91	95.07%±1.06	95.73%±1.81	95.28%±0.88	0.96±0.02

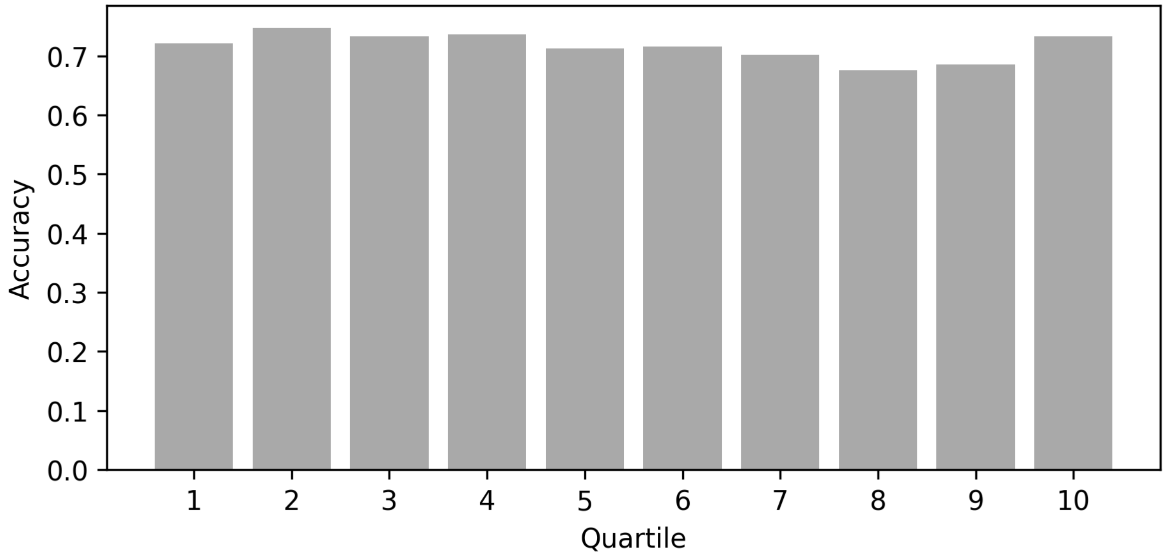

Supplementary Figure 4 | Accuracy on the test set prediction divided per quartile according to the average similarity score between test and training compounds in the VirtuousMultiTaste dataset.

Data curation

Supplementary Table 7 | Summary of the collected compounds for the initial dataset from the selected taste databases.

Reference	Taste	Number
**Biochemical Targets of Plant Bioactive Compounds by Gideon Polya** [1]	Bitter	39
	Sweet	32
**BitterDB** [2]	Bitter	1041
**Fenaroli Handbook of Flavor Ingredient** [3]	Bitter	33
	Sweet	426
	Tasteless	3
**Rodgers et al. (2006)** [4]	Bitter	29
**Rojas et al. (2017)** [5]	Bitter	81
	Sweet	435
	Tasteless	133
**SuperSweet** [6]	Sweet	397
**ToxNet** [7]	Tasteless	72
**The Good Scents Company Database**	Bitter	43
	Sweet	158
**Dagan-Wiener et al. (2017)** [8]	Bitter	100
**SweetenersDB** [9]	Sweet	316
**UMP442** [10]	Umami	140
**ChemTastesDB** [11]	Sweet	977
	Bitter	1183
	Umami	98
	Sour	38
	Salty	12
	Non-Sweet	233
	Tasteless	203
	Miscellaneous	87

Supplementary Table 8 | Summary of the refined dataset employed to train and test the model.

Class	#	References
Sweet	1904	VirtuousSweetBitter [12] + ChemTastesDB [11]
Bitter	1937	VirtuousSweetBitter [12] + ChemTastesDB [11]
Umami	227	UMP442 [10] + ChemTastesDB [11]
Other	649	Previous literature [3,7,13] + ChemTastesDB [11]

Supplementary Table 9 | Summary of the final dataset used in the present work.

Set	Class	Number
**Training**	bitter	360
	sweet	360
	other	360
	umami	227
**Test**	bitter	1577
	sweet	1544
	other	289
	umami	-

References

1. Polya, G. *Biochemical Targets of Plant Bioactive Compounds*. *Biochemical Targets of Plant Bioactive Compounds* (CRC Press, 2003). doi:10.1201/9780203013717.
2. Dagan-Wiener, A. *et al.* BitterDB: taste ligands and receptors database in 2019. *Nucleic Acids Res.* **47**, D1179–D1185 (2019).
3. Burdock, G. A. *Fenaroli's Handbook of Flavor Ingredients*. *Fenaroli's Handbook of Flavor Ingredients* (CRC Press, 2004). doi:10.1201/9781420037876.
4. Rodgers, S., Glen, R. C. & Bender, A. Characterizing bitterness: Identification of key structural features and development of a classification model. *J. Chem. Inf. Model.* **46**, 569–576 (2006).
5. Rojas, C. *et al.* A QSTR-based expert system to predict sweetness of molecules. *Front. Chem.* **5**, 1–12 (2017).
6. Ahmed, J. *et al.* SuperSweet-A resource on natural and artificial sweetening agents. *Nucleic Acids Res.* **39**, D377–D382 (2011).
7. ToxNet.
8. Dagan-Wiener, A. *et al.* Bitter or not? BitterPredict, a tool for predicting taste from chemical structure. *Sci. Rep.* **7**, 12074 (2017).
9. Chéron, J. B., Casciuc, I., Golebiowski, J., Antonczak, S. & Fiorucci, S. Sweetness prediction of natural compounds. *Food Chem.* **221**, 1421–1425 (2017).
10. Charoenkwan, P., Yana, J., Nantasenamat, C., Hasan, M. M. & Shoombuatong, W. IUmami-SCM: A Novel Sequence-Based Predictor for Prediction and Analysis of Umami Peptides Using a Scoring Card Method with Propensity Scores of Dipeptides. *J. Chem. Inf. Model.* **60**, 6666–6678 (2020).
11. Rojas, C. *et al.* ChemTastesDB: A curated database of molecular tastants. *Food Chem. Mol. Sci.* **4**, 100090 (2022).
12. Maroni, G. *et al.* Informed classification of sweeteners/bitterants compounds via explainable machine learning. *Curr. Res. Food Sci.* **5**, 2270–2280 (2022).
13. Rojas, C. *et al.* Quantitative structure–activity relationships to predict sweet and non-sweet tastes. *Theor. Chem. Acc.* **135**, 1–13 (2016).
